# Assessment of platelet indices and platelet activation markers in children with *Plasmodium falciparum* malaria

**DOI:** 10.1186/s12936-020-03218-4

**Published:** 2020-04-08

**Authors:** Renate Asare, Clement Opoku-Okrah, Kwabena Owusu Danquah, Ohene Opare-Sem, Otchere Addai-Mensah, Daniel Gyamfi, Francis Agyei Amponsah, Edward Y. Afriyie, Richard Vikpebah Duneeh, David Ntiamoah Ofosu, Michael Frimpong

**Affiliations:** 1 Heamatology Unit, Komfo Anokye Teaching Hospital (KATH), Kumasi, Ghana; 2 Department of Medical Laboratory Technology, Kwame Nkrumah University of Science and Technology (KNUST), Kumasi, Ghana; 3 Kumasi Center for Collaborative Research in Tropical Medicine (KCCR), Kumasi, Ghana; 4 St. John of God Hospital, Sunyani, Ghana

**Keywords:** Platelet membrane glycoproteins, Platelet indices, *Plasmodium falciparum*, Flow cytometry

## Abstract

**Background:**

*Plasmodium falciparum* malaria remains one of the world’s major infectious diseases that cause most morbidity and mortality, particularly in children. In Ghana, most children below the ages of 5 years depending on the severity of the infection often lose their lives. However, it is still debatable why infection with falciparum malaria contributes to thrombocytopenia.

**Methods:**

This study sought to investigate the expression of the various platelet indices and activation markers in children with falciparum malaria. Platelet indices (Platelet count [PLT], Plateletcrite [PCT], Mean Platelet Volume [MPV], Platelet Distribution Width [PDW] and Platelet-Large Cell Ratio [P-LCR]) and platelet surface membrane glycoproteins (GPIIb/IIIa [PAC-1], P-selectin [CD62p] and GPIV [CD36]) expressions were determined in children with falciparum malaria (cases) and healthy children (controls) using automated blood cell analysis and flow cytometry techniques, respectively.

**Results:**

Except for P-LCR, all the other platelet indices (PLT, MPV, PDW, and PCT) were significantly lower in the cases than the controls (P < 0.05). Also, it was observed that the level of expression of the activation markers; PAC 1 and CD62p showed a significant (P < 0.05) decreased before and after activation in falciparum malaria cases than in the controls. On the contrary, CD36 expression in the controls did not differ significantly (p > 0.05) from the malaria cases. Platelet activation markers were known to be associated with increased risk of *f*alciparum malaria with the mean fluorescence intensity of PAC1 (Odds Ratio [OR] 34.0, Relative Risk [RR] 4.47, 95% Confidence Interval [CI] 4.904–235.7; p < 0.0001) and CD36 (OR 4.2, RR 1.82, 95% CI 0.9824–17.96; p = 0.04). Moreover, the percentage expression of CD62p (OR 4.0, RR 1.80, 95% CI 0.59–27.24; p = 0.19) was also observed to be probably associated with increased risk of falciparum malaria although not statistically significant (p > 0.05).

**Conclusion:**

*Plasmodium falciparum* malaria has been known to be associated with platelet activation markers, which probably contributes to thrombocytopenia.

## Background

*Plasmodium* *falciparum* is the most prevalent malaria parasites on the African continent, responsible for most malaria deaths globally. It contributes to most childhood parasitic infection and presents with signs and symptoms such as recurrent fever, fatigue, and body joint pains. Other symptoms may include headache, nausea, chills, sweating, pallor and body weakness. Children with severe malaria frequently develop one or more of the following symptoms: severe anemia, respiratory distress, cerebral malaria [1] or thrombocytopenia.

Thrombocytopenia is a common haematological findings in children with malaria [2, 3] and the severity reflects increased parasite density. It might occur through peripheral destruction [4], excessive removal of platelets by splenic pooling [5, 6] as well as platelet consumption by the process of disseminated intravascular coagulopathy [DIC] [7]. Immune-mediated destruction of circulating platelets has also been postulated as a possible cause of thrombocytopenia in falciparum malaria. This might probably be due to elevated level of specific immunoglobulin G (IgG) in the circulatory system which binds to the platelet-bound malaria antigens [8]. Also, platelet activation molecules (GPIV, P-selectin and GPIIb/IIIa) have been reported to mediate clumping of parasitized red blood cells (PRBC) which contributes to increase sequestration and disease severity [8].

*Plasmodium falciparum* malaria reported as a common cause of thrombocytopenia [9] may also leads to platelet activation. Although most studies have demonstrated platelet interaction with parasitized red blood cells (PRBC), little is known about it association with platelet activation markers. This study, therefore, sought to assess the various platelet indices and platelet activation markers in children with falciparum malaria at Komfo Anokye Teaching Hospital.

## Methods

### Inclusion and exclusion criteria

Children between the ages of 2–16 years diagnosed of falciparum malaria and whose parents had consented were included in the study. Those on antiplatelet agents and reported abnormal prothrombin and activated partial thromboplastin time were excluded.

### Patients and normal controls participants

Following ethical approval, an unmatched case control study involving a total of 34 children between the ages of 2–16 years were recruited. Out of the 34 children, 19 were infected with *Plasmodium falciparum*. Fifteen were healthy children with no current history of malaria and bleeding episodes. Rapid diagnostic test using first response test kits and microscopic examination were used for the diagnosis of *falciparum* malaria.

### Sample collection and experimental approach

Six millilitres of whole blood were withdrawn from the study participants. A volume of 3.6 ml of blood was dispensed in the sodium citrate vacutainer tube and the remaining in the ethylenediamine tetraacetic acid (K_2_ EDTA) sample tube (Beckton-Dickson [BD]). Sodium citrate blood samples were used for flow cytometry analysis of the platelet membrane glycoproteins. Venous blood samples collected into anticoagulant EDTA- K_2_ sample tubes were also analysed for the assessment of the various platelet indices.

### Platelet activation

Platelet membrane glycoproteins were detected following the protocol described by Becton Dickson (BD) [10]. Sodium citrate blood samples were processed within 20 min of blood collection. Platelets were activated by using 50 mg/mL adenosine diphosphate solution (ADP) [Sigma Aldrich, USA].

### Detection of platelet membrane glycoproteins

Ten microlitres of platelet-specific antibodies (PAC-1-FITC, CD62p-PE and CD36-PE [Becton Dickson (BD), USA]) were used in the detection of the platelet membrane glycoproteins (GPIIb/IIIa, P- Selectin and GPIV respectively). PAC1- FITC and CD62p-PE were double stained while CD36- PE was single stained. 5μL Arginylglycylaspartic acid (RGD) [Sigma—Aldrich, USA] solution was added in the staining mixture of PAC-1-FITC and CD62p-PE to demonstrate specific PAC-1 binding.

Equal volumes (2.5 μL) of inactivated and activated blood samples were stained with the antibodies and incubated for 15 min at room temperature in the dark. 500 μL of the cold 1% paraformaldehyde solution (BD, USA) was used to fix the stained cells for flow cytometry analysis.

### Analysis by flow cytometry

**Fig. 1:**
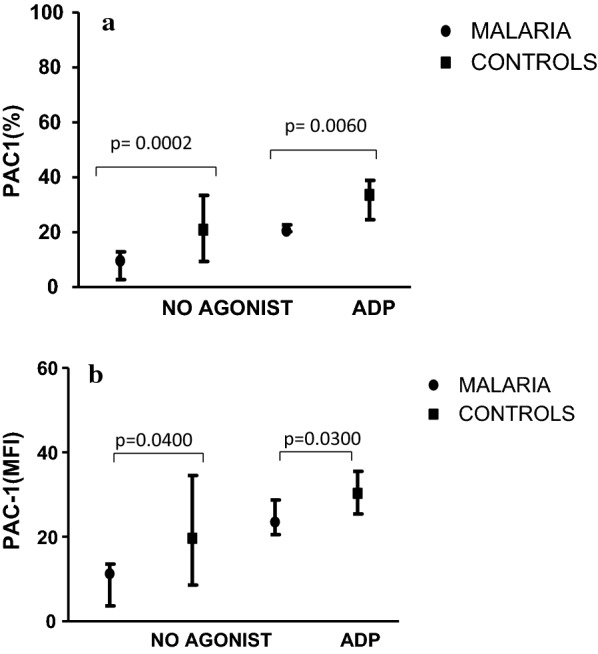
**a** Percentage (%) expression of PAC1 (Resting and activation phase). Expression of PAC1 (%) before (No agonist) and after (ADP) activation in controls and malaria cases. Median (25th and 75th percentile) shown. **b** Mean Fluorescence Intensity (MFI) expression of PAC1 (Resting and activation phase). MFI expression of PAC1 before (No agonist) and after (ADP) activation in controls and malaria cases. Median (25th and 75th percentile) shown

Acquisition and analysis of the processed samples were performed on scatter gating using CellQuestPro software of the FasCalibur (BD). The flow cytometry settings or acquisition of platelets were optimized by changing the linear scale to logarithmic signal amplification in all five detectors. FacsComp was used in daily CaliBrite beads calibration. A total of 10,000 events in a log side scatter versus forward scatter were collected for each sample. The analysis of the platelet activation markers PAC1- FITC, CD62p- PE and CD36-PE were based on the gated population enclosing platelets as defined by forward scatter (FSC) and side scatter (SSC) characteristics (Fig. 1). To avoid non-specific binding, the threshold was set using the cells stained with isotype control antibody or RGDS. The platelet fluorescence of the activation markers were measured as percentage platelet expression (%) and mean florescence intensity (MFI).

### Detection of platelet-related parameters (PLT, MPV, PDW, PCT, and P-LCR)

Ethylendiaminetetraacetic acid (EDTA-K_2_) blood samples were analysed using the Sysmex XT4000i haematology analyzer to assess the various platelet indices.

### Bleeding score assessment

Bleeding status of children with falciparum malaria were assessed using a standardized bleeding scale. This scale measures bleeding at six different anatomic sites (i.e. epistaxis, cutaneous bleeding, oral cavity bleeding, gastrointestinal bleeding, haematuria and bleeding from minor wounds or surgery). Bleeding score was later summarized into bleeding and no bleeding, the type of bleeding and its corresponding score. The bleeding symptoms was scored from grade 0 (absence or no symptoms) to grade 3 (symptoms requiring medical intervention) and the overall bleeding score was achieved by adding the scores for all of the bleeding symptoms [11].

### *Plasmodium falciparum* species identification

The identification of *Plasmodium falciparum* species was first performed using the first response rapid diagnostic test kit (RDT), and finally confirmed with microscopy. Five microlitres of EDTA- K_2_ blood samples were placed in the sample wells using the pipettes. The assay diluent was completely emptied by dispensing it to the diluent wells and the results were read within 20 min. Thin and thick blood films were prepared and flooded with Giemsa. The stained slides were washed under running tap water after 10 min. The stained blood films were allowed to air dry and viewed microscopically using × 100 (oil immersion) objective lens.

### Detection of prothrombin (PT) and activated partial thromboplastin time (APTT)

Sodium citrate anticoagulant blood samples were spun for 15 min after sample preparation for flow cytometry. Fifty microlitres of plasma were used for the measurement of PT and APTT using CoaRad 2A (Axiom, Germany).

### Statistical analysis

The data obtained in this study was analysed using EPI v7.2.0.1 info (CDC) and Graphpad prism 5. All the categorical variables and continuous variables were summarized by descriptive analysis. Continuous data was expressed as median (25th and 75th percentile range and interquartile ranges) for skewed data and the difference between the various groups was tested using the student *t* test (Mann–Whitney). The effect of the categorical variables on the possible disease outcome was estimated using the Odds ratio (OR) with 95% confidence intervals (CI) for multivariate and univariate analysis. The various proportions were assessed using Fisher’s-exact or X^2 −^ test. A *p* value < 0.05 was considered statistically significant.

## Results

### Comparison of ages and gender among the cases and controls

Table 1 summarized the ages and gender among the study participants (cases (Malaria infected children) and controls [healthy children]). The median age of malaria group [3.0 (2.0–5) years] was statistically significantly (p > 0.05) lower than the controls [11.0 (8.0–12.0) years] as shown in Table 1. In addition, females were more than males in both cases and controls.

**Table 1:** Comparison of age and gender among the three groups

Study groups	Sample size	Gender		Median age (years)^a^
		Male	Female	
Cases (Malaria infected children)	19	8	11	3.0 (2.0–5)
Controls (Healthy children)	15	7	8	11.0 (8.0–12.0)
Total	34	15	19	
				p-value = 0.025

^a^Data are presented as medians, with 25th and 75th inter-quartile ranges in parentheses

### Bleeding score assessment

Three children infected with P. *falciparum* malaria had bleeding history which was graded as minor type (grade 1–2). None of the participants had a bleeding history of grade 3–4. The commonest bleeding type reported in the cases include oral bleeding, haematuria and epistaxis (Table 2). However, bleeding was not statistically significantly associated with *P. falciparum* malaria (odds ratio [OR] 0.76, 95%CI 0.11–5.23; p = 0.58).

**Table 2:** Bleeding score assessment of the three groups

Bleeding tendencies	Controls	Cases
Bleeding (Y/N)^a^	0/15	3/19
No bleeding (Y/N)^a^	15/15	16/19
Bleeding type	None	Oral, Epistaxis, Haematuria
Bleeding score	Grade 0	Grade 1

^a^Y/N means Yes/No

### Detection of platelet indices

The median (25th and 75th percentiles) expressions of the platelet indices (PLT, PCT, PDW, P-LCR and MPV) in children with falciparum malaria were lower compared to the controls. However, with the exception of the P- LCR, all the other platelet indices recorded in the cases differed statistically significant from the controls (Table 3).

**Table 3:** Expression of platelet indices among the malaria cases and control groups

Platelet indices	Cases^a^	Controls^a^	P-value
PLT (10^3/µL)	106 (65–205)	197 (169–237)	0.030
PCT (%)	0.08 (0.00–0.18)	0.23 (0.18–0.26)	0.003
MPV (L)	10.00 (0.00–11.20)	11.10 (9.90–12.90)	0.030
PDW (fL)	11.00 (0.00–12.50)	13.30 (11.00–16.40)	0.020
P-LCR (%)	25.00 (0.00–32.80)	29.50 (22.80–36.90)	0.130

^a^Data are presented as medians, with 25th and 75th inter-quartile ranges in parentheses

### Detection of the percentage (%) and mean fluorescence Intensity (MFI) expression of platelet membrane glycoproteins during resting and activation phase

#### Expression of PAC 1 analysis

The percentage expression of PAC -1 was significantly (p < 0.05) lower in children with falciparum malaria (9.66 [2.81–12.86]%) than the controls (20.94 [9.34–33.48]%) in both the resting and activation phase. However, there were an increase expression of PAC-1 in both the malaria group (20.56 [20.19–22.74]%) and the controls (33.62 [24.66–38.86]%) during the activation phase. But the expression still remained significantly (p < 0.05) higher in the controls than in the cases (Fig. 1a). The same trend was again observed in the mean fluorescence intensity (MFI) of PAC-1 expression (Fig. 1b).

#### Expression of CD62p analysis

**Fig. 2:**
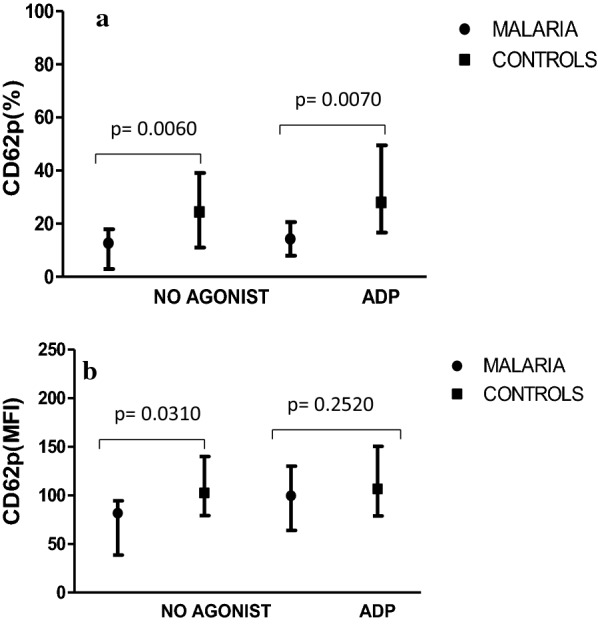
**a** Percentage (%) expression of CD62p (Resting and activation phase). Expression of CD62p (%) in controls and malaria cases before (No agonist) and after (ADP) activation in controls and malaria cases. Median (25th and 75th percentile) shown. **b** Mean Fluorescence Intensity expression of CD62p (Resting and activation phase). MFI expression of CD62p before (No agonist) and after (ADP) activation in controls and malaria cases. Median (25th and 75th percentile) shown

The expression of CD62p was significantly lower in children with malaria (12.68 [2.98–17.98]%) than the healthy children (24.48 [11.05–39.10]%) during the resting phase (Fig. 2). After the addition of the agonist, the expression of CD62p significantly increased in the controls (28.06 [16.67–49.56]%) than in the cases (14.30 [7.92–20.60]%). On the contrary, no significant difference was detected in the activation phase in the MFI expression of CD62p.

#### Expression of CD36 analysis

**Fig. 3:**
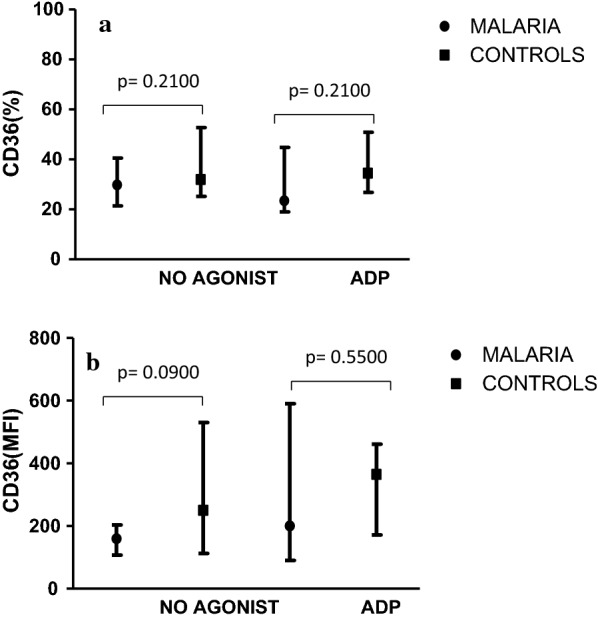
**a** Percentage (%) expression of CD36 (Resting and activation phase). Expression of CD36 (%) in controls and malaria cases before (No agonist) and after (ADP) activation in controls and malaria cases. Median (25th and 75th percentile) shown. **b** Mean Fluorescence Intensity expression of CD36 (MFI). MFI expression of CD36 in controls and malaria cases before (No agonist) and after (ADP) activation in controls and malaria cases. Median (25th and 75th percentile) shown

There was no significant difference in the expression of CD36 among the cases (29.75 [21.34–40.57]%) and the controls (31.92 [25.10–52.71]%) during the resting phase (Fig. 3a). Moreover, at the activation phase, CD36 showed increased expression in the controls (34.44 [27.75–50.91]%) but decreased in the malaria groups (23.49 [18.98.70–44.75]%) without any significant difference (Fig. 3a). Similar results was again observed in the mean fluorescence intensity (Fig. 3b).

### Establishing a link between falciparum malaria and platelet activation markers

An association was established between *P. falciparum* malaria and mean fluorescence intensity expression of the platelet activation markers. Thrombocytopenia was known to be associated with increased risk of *P*. *falciparum* malaria with the MFI of PAC1 (Odds Ratio [OR] 34.0, Relative Risk [RR] 4.47, 95% Confidence Interval [CI] 4.904 to 235.7; p < 0.0001) and CD36 (OR 4.2, RR 1.82, 95% CI 0.9824 to 17.96; p = 0.04) (Table 5). Again, a link was established between falciparum malaria and the median percentage of the activated markers. Although no significant difference (p > 0.05) in the percentage expression of CD62p was recorded, increased risk of falciparum malaria might also be associated with CD62p as shown in Table 4.

**Table 4:** Association between *P. falciparum* malaria and (%) expression of the platelet activation markers

Platelet activation markers	Odds ratio (OR)	Confidence interval (95% CI)	P-value (p > 0.05)
PAC 1	0.96	0.15–5.90	0.66
CD 62p	4.00	0.59–27.24	0.19
CD36	0.88	0.13–5.82.40	0.58

## Discussion

Falciparum malaria is a common parasitic infection in children under 5years living in endemic areas. It was observed in this study (Table 1), that most of the malaria cases had decreased ages than the controls. The difference in the ages might be due to low immunity in children below the ages of 6 years, contributing to the high susceptibility of malaria infection. The decreased median age (3.0 years) in the cases was contrary to Murray et al. [12], who reported an increasing prevalence of falciparum malaria in children under 15 years. This might be due to differences in geographical location. However, it was by the records of the Ministry of Health Ghana [13], stating that the highest incidence and fatality rate of falciparum malaria are common in children below 5 years [13].

From the study, most children with falciparum malaria had low platelet counts compared to the controls. PLT is a dynamic indicator of peripheral blood platelet production and destruction. This was used in this study to assess thrombocytopenia in children with falciparum malaria. The significant decrease in PLT counts (Table 3) in the malaria cases somehow explained the link between platelets and *P*. *falciparum* species. Studies have proven that platelets protect the host during erythrocytes infection by releasing platelet factor 4 (PF4) molecules which has plasmocidal activity [14, 15]. As a result, more platelets are utilized during the release of PF4 in response to increased parasitized red cells (pRBCs). This, therefore, probably leads to the increased platelet destruction leading to thrombocytopenia. Besides, there were also significantly decreased levels in the expression of Plateletcrit (PCT) (Table 3) in the malaria cases than the controls. This was probably due to the low PLT level. PCT is the volume occupied by platelets in the blood and the changes in PCT are generally consistent with the changes in PLT. Except for the P-LCR, the remaining platelet indices (PDW and MPV) detected were significantly lower in cases than the controls (Table 3). The various platelet parameters (PDW and MPV) are the best indicators to assess platelet activity or functionality. Thus, the low levels of these indices might be associated with it functionality.

To assess platelet functionality, flow cytometry analysis was performed using PAC-1, CD62p and CD36 monoclonal antibodies for the detection of GPIIb/IIIa, P-selectin and GPIV, respectively. Except for CD36, the expression of the other platelet markers in malaria cases differed significantly from the controls. Also, a decreased expression of the platelet membrane glycoproteins was observed in the malaria cases than in the controls. This may be due to the low level of circulatory platelets as a result of immune-mediated destruction of the platelets. Alternatively, increased splenic removal of platelet clumped PRBC or increased release of platelet factor 4 (PF4) molecules may also be a contributory factor. The increased expression of the membrane glycoproteins (Figs. 1, 2) after activation in the malaria cases might suggest normal platelet functionality as demonstrated by Gérardin et al. [16]. His study proved that activated platelets in malaria-infected patients are hypersensitive and enhance haemostatic responses. This was in agreement with Inyang et al. [17] and Srivastava et al. [18] who observed an enhanced platelet aggregation and increased surface expression of CD62p and PAC-1 after incubating platelets with pRBCs. The normal platelet functionality might be one of the reasons we reported a low number of bleeding tendencies. Three children with falciparum malaria had bleeding episodes (Table 2) during the bleeding assessment. They presented with oral bleeding, haematuria, and epistaxis (grade 1 type).

Platelet activation markers (PAC1 and CD36) were observed to be significantly (p > 0.05) associated with increased risk of falciparum malaria (Table 5). Furthermore, the percentage expression of CD62p might also be associated with increased risk of falciparum malaria although not statistically significant (p > 0.05) (Table 4). Glycoprotein IV (GP IV) has been reported to induce clumping of *P*. *falciparum*- pRBCs in vitro leading to pathogenesis of cerebral malaria [8]. This was in agreement with Wassmer et al. [8] who reported CD36 and P-selectin as the major platelet molecules responsible for cerebral malaria in Malawian children. Thus, the low level of CD36 after activation in this study, was probably an indication of impairment of the normal function of GP IV.

**Table 5:** Association between *P*. *falciparum* malaria and MFI expression of the platelet activation markers

Platelet activation markers	Odds ratio (OR)	Confidence interval (95% CI)	P-value (p > 0.05)
PAC 1	34.0	4.904–235.7	0.0001
CD 62p	1.60	0.7333–1.838	0.19
CD36	4.20	0.9824–17.96	0.04

## Conclusion

In summary, falciparum malaria has been known to be associated with the various platelet activation markers which possibly contribute to thrombocytopenia. Severe malaria contributes to decrease platelet surface membrane glycoproteins due to excessive adhesion of platelets to parasitized red blood cells. Hence, further studies are recommended in establishing the mechanism of the various platelet membrane glycoproteins in the pathogenesis of falciparum malaria.

## Data Availability

The datasets used and/or analysed during the current study are available from the corresponding author on reasonable request.

## Abbreviations

APTT: Activated partial thromboplastin time
CD: Cluster of differentiation
DIC: Disseminated intravascular coagulopathy
EDTA: Ethylene diamine tetra acetic acid
FC: Flow cytometry
FITC: Fluorescein isothiocyanate
FSC: Forward scatter
GP: Glycoprotein
IgG: Immunoglobulin G
PAC-1: Procaspase-activating compound 1
PRBC: Parasitized red blood cell
PCT: Plateletcrite
PF4: Platelet factor 4
PDW: Platelet distribution width
PE: Phycoerythrin
PLT: Platelet count
P-LCR: Platelet large cell ratio
PT: Prothrombin time
RDT: Rapid diagnostic testing
SSC: Side scatter
WHO: World Health Organization
